# Should We Bandage after Labor?

**Published:** 1880-07-20

**Authors:** Alexander Elwell

**Affiliations:** Vincentown, N. J.


### SHOULD WE BANDAGE AFTER LABOR, AND WHY? AND IF NOT, WHY NOT?

BY ALEXANDER ELWELL, M. D., VINCENTOWN, N. J.

These questions have presented themselves to my mind, from the fact that I have found a growing disposition on the part of some of my brethren to break in upon the long and useful custom of bandaging our parturient patients. Not long since, I called to my assistance, in a case of labor, a gentleman whom I have always held in high esteem, both for his medical opinion as well as his gentlemanly and social qualities, who, upon the question about the bandage, remarked that it was of but little importance whether she was bandaged or not; this set me to thinking over the subject, a subject that Prof. Hodge in his lectures laid much stress upon, and which other authors, such as Dewees, Meigs, James and Ramsbotham, had led me to think was of very much importance. The disciples of Hannheman do not bandage, but I see no cause why we should follow in their footsteps. The principal object which the bandage serves is to brace the bowels, and give artificial support in place of that which they have lost through the extreme laxity of the abdominal muscles, and to prevent the faintness frequently attendant on the sudden removal of a certain degree of pressure. I also think that a bandage, properly applied, stimulates the uterus to contraction; though of course too much reliance must not be placed on it for that purpose, but the uterus should be induced to contraction before the application of the bandage by gentle and continued friction over and to it. Again, I think the bandage serves other purposes—such as the prevention of anteversion, lateral obliquities, and even prolapsis—by relieving the uterus of the weight of over-distended muscles and dislocated intestines, until nature and time comes to the relief, giving tone and strength to surroundings. My idea of a bandage is one that is wide enough to come from the ensiform cartilage well down over the hips, with gussets or gores, fitting smoothly, showing but little disposition to work up; it should be worn from six to eight weeks; then we will find our patients to walk with more erect, firm and elastic step, complaining less with backache and headache and general lassitude. A bandage thus made and worn, with the addition of two or three napkins under it, next to the skin, will keep us advised of any tendency to hemorrhage, should it exist.

I am certain I have seen flooding where there was no importance attached to bandaging or none applied, that might have been avoided in part, or perhaps altogether. Sometimes, in hasty delivery, the birth occurring before the arrival of the accoucher, the patient being allowed to lie without the proper attention, concealed hemorrhage does occur. One such case comes very vividly to my mind. I was sent for, and upon my arrival I found, to my great astonishment, my patient dead. The child was born, and the placenta self-delivered. Upon questioning those around, no satisfactory answer could I get as to the supposed cause of this sudden taking off. Had there been much flooding? I asked. Not more that usual in cases of confinement. I was not satisfied, and made an examination post mort, and found the uterus filled with clotted blood, and as large as at the seventh month of pregnancy. Now, had the uterus been held firmly in place by bandage and napkins, I cannot help thinking that much of the flooding might have been avoided, and the patient's life saved. Other similar cases I have seen, and am led to believe that the timely use of the bandage, and preceding manipulations, saved my patient. So thoroughly am I convinced of their usefulness, I would not dare to leave off their use.

I have endeavored to give some reasons why the bandage should be used, and the negative side of the question I leave to those to answer that may differ from me. Should there be good reasons given why bandaging may or should be done away with, then would I consider we are rid of very much trouble.—*Country Practitioner.*

[A bandage is useful in giving support to the empty and relaxed condition of the abdominal walls, and may, by its pressure, tend in some measure to maintain the womb in a state of contraction; but we have found from experience that it should not be hastily applied, as usually advised, because it interferes with the manipulation necessary to detect contraction and guard against post partum hemorrhage.—ED. RECORD–W.

